# Elacestrant in hormone receptor-positive metastatic breast cancer: a post-hoc analysis

**DOI:** 10.37349/etat.2025.1002293

**Published:** 2025-02-20

**Authors:** Azza Sarfraz, Muzna Sarfraz, Faheem Javad, Musfira Khalid, Bushra Shah, Amna Gul, Mohammad Arfat Ganiyani, Areeba Ismail, Khadija Cheema

**Affiliations:** IRCCS Istituto Romagnolo per lo Studio dei Tumori (IRST) “Dino Amadori”, Italy; ^1^Department of Pediatrics, The Aga Khan University, Karachi 74800, Pakistan; ^2^Department of Research, King Edward Medical University, Lahore 54000, Pakistan; ^3^Department of Research, Al Nafees Medical College, Islamabad 45600, Pakistan; ^4^Department of Research, Fatima Jinnah Medical University, Lahore 54000, Pakistan; ^5^Department of Research, Liaquat National Medical College and Hospital, Karachi 74800, Pakistan; ^6^Department of Medical Oncology, Miami Cancer Institute, Baptist Health South Florida, Miami, FL 33176, USA; ^7^Department of Research, Jinnah Sindh Medical University, Karachi 75510, Pakistan; ^8^Department of Medicine, Tucson Medical Center, Tucson, AZ 85712, USA

**Keywords:** Elacestrant, selective estrogen receptor degraders, breast cancer, antineoplastic agents, hormone receptor-positive

## Abstract

**Background:**

Breast cancer is a leading cause of mortality in women. Hormone therapy plays a crucial role in treatment of hormone receptor-positive metastatic breast cancer. Elacestrant is a selective estrogen receptor degrader (SERD) that has shown promise in early-phase clinical trials. This post-hoc analysis systematically evaluates elacestrant’s effectiveness in hormone receptor-positive metastatic breast cancer patients, providing insights into its efficacy, safety, and potential advantages over existing treatments.

**Methods:**

We adhered to the PRISMA Statement 2020 guidelines and systematically searched the databases PubMed/MEDLINE, ClinicalTrials.gov, Web of Science, and Embase. We conducted the post-hoc analysis using R software (V 4.3.3), applying the inverse variance method and the DerSimonian-Laird estimator to pool effect estimates with a random-effects model. We assessed heterogeneity using the Cochran’s Q test and the *I*^2^ statistic.

**Results:**

Our post-hoc analysis encompassed 3 clinical trials and a total of 835 participants. The mean age of all 835 participants across the three trials was 59.5 years (95% CI: 58.7–60.3). The pooled progression-free survival (PFS)—was estimated at 4.38 (95% CI: –7.58–16.35, *P* = 0.47), and the pooled objective response rate (ORR) was 7% (95% CI: 2–18%, *P* = 0.04), with significant heterogeneity observed among the studies.

**Discussion:**

Elacestrant shows promise for improving outcomes in hormone receptor-positive metastatic breast cancer, but further research is needed to confirm its effectiveness. Future studies should include larger sample sizes, comprehensive phase II and III trials, and investigation of elacestrant in combination with other drugs or in preoperative settings.

## Introduction

Breast cancer is the second leading cause of death among women worldwide, impacting approximately 260,000 individuals and resulting in 40,000 deaths annually [1]. Over two-thirds of these cases are classified as hormone receptor-positive breast cancer [2]. Affecting about one in eight women during their lifetime, breast cancer is one of the most prevalent cancers diagnosed in women. Although rarer in men, the incidence of breast cancer in males is increasing, with contributing factors including Klinefelter syndrome, high body mass index, testicular and liver diseases, radiation exposure, and alcohol consumption [3]. Additionally, about 10–15% of breast cancer patients develop brain metastases, typically appearing 2–3 years after the initial diagnosis [1].

In patients with estrogen receptor-positive (ER+) metastatic breast cancer, hormone therapy remains the primary treatment option to delay the need for chemotherapy [4]. The current standard of care (SOC) for ER+ metastatic breast cancer involves a combination of hormone therapy and CDK4/6 inhibitors [5]. However, resistance often arises due to mutations in the *ESR1* gene, which encodes the estrogen receptor [6]. For patients who progress despite hormone therapy and CDK4/6 inhibitors, sequential endocrine monotherapy is typically recommended. Fulvestrant, approved by the U.S. Food and Drug Administration (FDA) in 2002, is administered via intramuscular injection and has shown better efficacy than tamoxifen and aromatase inhibitors (AIs). It is commonly used as a second- or third-line treatment [7]. Despite its effectiveness, fulvestrant is associated with a relatively low median progression-free survival (PFS) of just 2 months. Additionally, most patients eventually develop resistance to the drug, although the precise mechanisms behind this resistance are not yet fully understood [8]. Furthermore, fulvestrant’s limited bioavailability has prompted the development of oral selective estrogen receptor degraders (SERDs), which offer the potential for improved bioavailability and effectiveness [9].

SERDs are antiestrogens designed to destabilize the H12 region of the estrogen receptor, working by binding to the receptor and promoting the degradation of the ER signaling pathway [10]. Several SERDs are currently in clinical development [7], with elacestrant (RAD-1901) being one of the notable non-steroidal small molecules that acts as an estrogen receptor antagonist [11]. On January 27, 2023, the U.S. FDA approved elacestrant for the treatment of advanced or metastatic breast cancer in patients with ER+, *ESR1*-mutated, and HER2-negative (HER2–) profiles, following progression after at least one line of endocrine therapy [12]. This approval marked elacestrant as the first oral estrogen receptor antagonist approved for patients with *ESR1* mutations [13]. Two phase I clinical trials (NCT02650817, NCT02338349) evaluated elacestrant in patients with hormone receptor-positive breast cancer that had metastasized to the brain [1]. With elacestrant now approved, patients with PIK3CA mutations and metastatic ER+ breast cancer may need to consider the benefits and risks of combining alpelisib and fulvestrant vs. using elacestrant as a single agent [7]. As elacestrant is integrated into standard care, molecular profiling will become increasingly important in treatment decisions, underscoring the critical role of precision medicine in managing breast cancer [14].

This post-hoc analysis aims to systematically evaluate the effectiveness of elacestrant as a therapeutic option in the management of breast cancer. By aggregating and analyzing data from clinical trials, we intend to provide a comprehensive understanding of the drug’s efficacy, safety, and potential advantages over existing treatments.

## Materials and methods

### Search strategy

**Figure 1:**
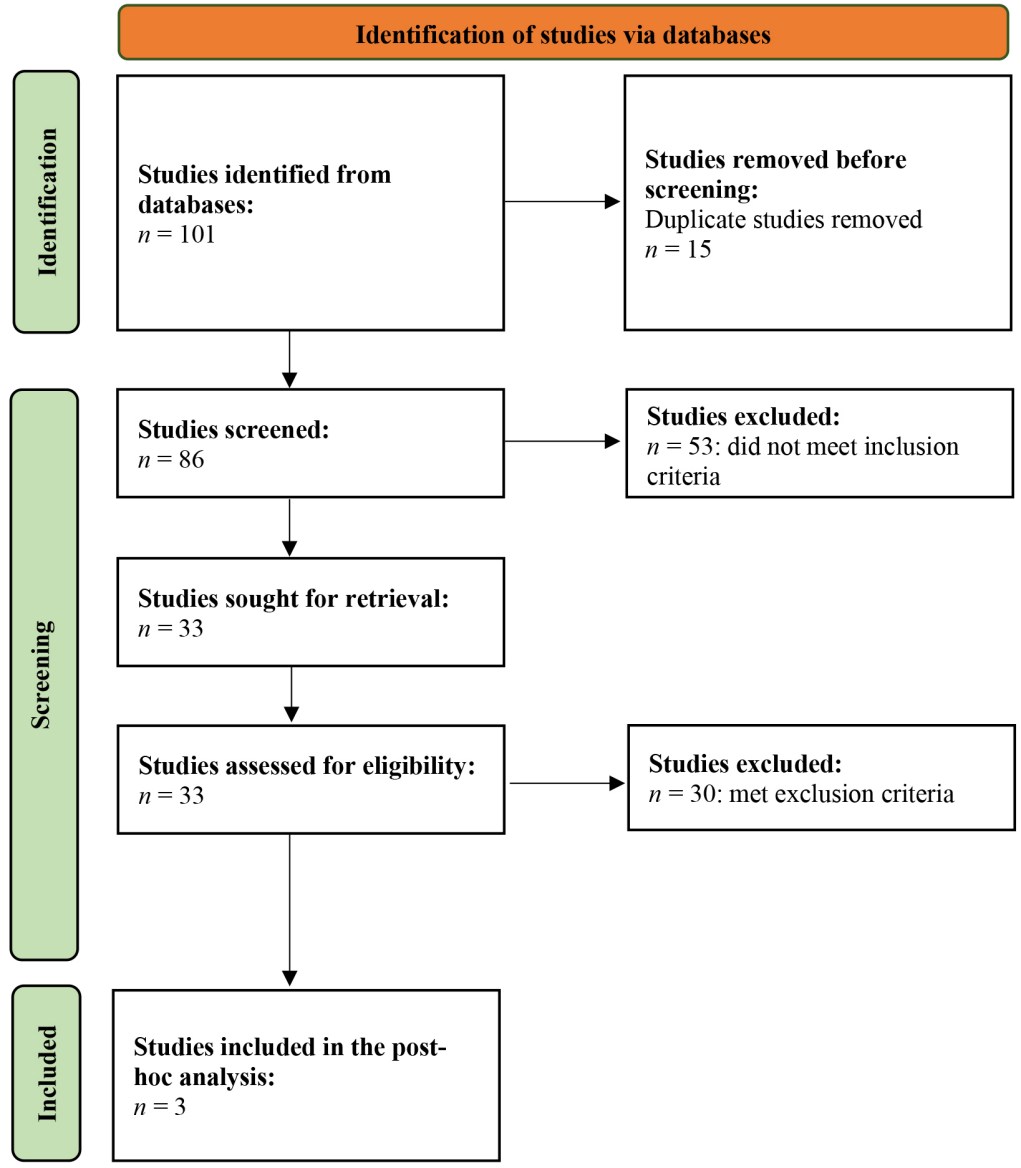
PRISMA flowchart depicting the study selection process

A comprehensive literature search was conducted following the PRISMA Statement 2020 guidelines [15] in the following databases: PubMed/MEDLINE, ClinicalTrials.gov, Web of Science, and Embase. In PubMed/MEDLINE, 28 published studies were obtained using specific keywords related to elacestrant and breast cancer. Similarly, 10 records were identified in ClinicalTrials.gov using relevant keywords. In Web of Science, 39 studies were retrieved, and in Embase, 24 results were found. The PRISMA flowchart is appended in Figure 1.

Keyword combinations for all the databases are as follows:

- PubMed/MEDLINE: (“elacestrant”[Supplementary Concept] OR “elacestrant”[All Fields]) AND (“breast neoplasms”[MeSH Terms] OR (“breast”[All Fields] AND “neoplasms”[All Fields]) OR “breast neoplasms”[All Fields] OR (“breast”[All Fields] AND “cancer”[All Fields]) OR “breast cancer”[All Fields]).
- ClinicalTrials.Gov: elacestrant and breast cancer.
- Web of Science: (elacestrant OR RAD1901) AND [breast AND (cancer OR neoplasm* OR carcinoma OR tumor OR tumour)].
- Embase: (elacestrant OR RAD1901) AND [breast AND (cancer OR neoplasm* OR carcinoma OR tumor OR tumour)].

The final search was conducted on 31st March, 2023 with no language restrictions. The population of interest consisted of patients with hormone receptor-positive breast cancer. The intervention under investigation was elacestrant treatment, while the comparator group included SOC or other treatments for hormone receptor-positive breast cancer. The outcomes assessed in the included studies were PFS, objective response rate (ORR), overall survival (OS), and adverse events (AE).

### Data extraction and synthesis

Data from eligible studies were extracted and tabulated according to the following variables: Author, Year, Study Design, Inclusion Criteria, Participant Count, Intervention Given, Previous Treatment, Primary Endpoints. For the datasheet extraction, the following information was included: Author, Year, Age, Previous treatment, PFS (%), ORR, OS (%), and AE (any).

### Statistical analysis

The post-hoc analysis was performed using R software. For PFS, the inverse variance method was employed to pool the effect estimates from individual studies, accounting for differences in sample sizes and variances. The DerSimonian-Laird estimator was used to estimate between-study variance (τ^2^) which represents the heterogeneity in the true effect sizes across studies. A random-effects model was applied to account for potential clinical and methodological diversity among the included studies. For ORR, the inverse variance method was used for pooling the effect estimates, similar to the PFS analysis. The DerSimonian-Laird estimator was applied to estimate τ^2^, and a random-effects model was used to account for heterogeneity. Additionally, the logit transformation was employed to stabilize the variances of the proportions, making the data more suitable for pooling. Clopper-Pearson confidence intervals were calculated for individual studies to account for the uncertainty in the estimated proportions. Heterogeneity among the included studies was assessed using the Cochran’s Q test and quantified using the *I*^2^ statistic.

## Results

### Overview of the included trials

Three clinical trials were included in this study exploring elacestrant as a therapeutic option for hormone receptor-positive breast cancer [16–18]. Although the design, goals, and outcomes of each study varied, all three aimed to assess the effectiveness and safety of elacestrant. The mean age of all 835 participants across the three trials was 59.5 years (95% CI: 58.7–60.3) years. AE (any) were reported in 282 of 303 participants (93%) in the elacestrant group [16–18], whereas in the standard care group of the phase III trial, 197 of 229 participants reported any AE [16]. The characteristics of the included trials are enlisted in Table 1.

**Table 1:** Characteristics of the included trials

**Author, year**	**Study design**	**Inclusion criteria**	**Participant count**	**Intervention given**	**Previous treatment**	**Primary endpoints**
Bidard et al. [16], 2022	Randomized, open-label, phase III trial (NCT03778931)	- Postmenopausal women or men - Age: 18 years or older - Histologically or cytologically proven ER+/HER2– breast adenocarcinoma - Either locoregionally recurrent or metastatic disease	477; elacestrant (*n* = 239), SOC (*n* = 238)	Elacestrant dosing: - 400 mg orally once daily - Reductions to 300 mg or 200 mg daily permitted for toxicity Standard of care (SOC) treatment: - Per investigator’s choice (fulvestrant, anastrozole, letrozole, or exemestane monotherapy) - Dosed according to the labeling	- CDK4/6 inhibitors (progression on previous treatment was required) in combination with fulvestrant or an AI - One chemotherapy regimen in the advanced/metastatic setting was permitted	- PFS in all patients, assessed by BICR using standard RECIST v1.1 criteria—PFS in patients with detectable *ESR1* mutation, assessed by BICR using standard RECIST v1.1 criteria
Bardia et al. [17], 2021	Multicenter, open-label, four-part, dose-escalation study (NCT02338349)	- Postmenopausal women, age ≥ 18 years - ER+ (≥ 1% staining by immunohistochemistry) - HER2− locally advanced, inoperable, and/or metastatic breast adenocarcinoma - ECOG performance status 0–1	50	The RP2D was 400 mg of elacestrant once daily	Part A–C: - Required ≤ 2 prior lines of chemotherapy for advanced/metastatic breast cancer - Required ≥ 6 months of prior ET (no limit) in any setting Part D: - Required ≤ 1 prior line of chemotherapy for advanced/metastatic breast cancer - Required ≥ 2 prior lines of ET for advanced/metastatic breast cancer (single agent/in combination) - Required one prior line of treatment with fulvestrant with documented progression and prior CDK4/6i	Frequency of DLTs during the first 28 days of treatment
Jager et al. [18], 2020	Phase 1b, open-label, non-randomized (NCT02650817)	- Postmenopausal women, prior bilateral ovariectomy - Histologically proven ER+ (defined as ≥ 1% staining by immunohistochemistry), HER2− ABC (either inoperable primary breast cancer or mBC) - ECOG performance status: 0–2	16	- Initially enrolled and treated with 400 mg elacestrant capsule once daily (QD) - Second cohort of 8 patients enrolled and treated with 200 mg elacestrant capsule QD for 14 days to assess target engagement of a lower dose -After 14 days, the dose was escalated to 400 mg QD	- 6 or more months of 1–3 lines of systemic endocrine treatment for mBC - Prior CDK4/6 inhibitor therapy were allowed	Percentage difference in FES uptake in tumor lesions (up to a maximum of 20 lesions) after 14 days of treatment with elacestrant compared to baseline

AI: aromatase inhibitor; BICR: blinded independent central review; DLTs: dose-limiting toxicities; ER+: estrogen receptor-positive; FES: fluoroestradiol; PFS: progression-free survival; RP2D: recommended phase 2 dose; HER2–: HER2-negative

In their 2022 phase III trial (NCT03778931), Bidard et al. [16] enrolled 477 participants with ER+/HER2– breast adenocarcinoma. The subjects were divided into two groups: one received elacestrant (400 mg daily), while the other received SOC treatment (fulvestrant, anastrozole, letrozole, or exemestane monotherapy). The study’s primary endpoints were PFS for all participants and for those with a detectable *ESR1* mutation, as assessed by a blinded independent central review (BICR) using RECIST v1.1 criteria.

In a 2021 multicenter, open-label, four-part, dose-escalation study (NCT02338349), Bardia et al. [17] included 50 postmenopausal women with ER+, HER2– breast cancer. The subjects received 400 mg of elacestrant once daily. The study aimed to establish the recommended phase 2 dose (RP2D) of elacestrant and evaluate the frequency of dose-limiting toxicities (DLTs) during the initial 28 days of treatment.

Jager et al.’s [18] 2020 phase 1b, open-label, non-randomized trial (NCT02650817) involved 16 participants with ER+, HER2– advanced breast cancer. Initially, patients were treated with 400 mg of elacestrant daily, while a second cohort received a lower dose of 200 mg daily for 14 days before increasing to 400 mg daily. The study’s primary endpoint was the percentage difference in fluoroestradiol (FES) uptake in tumor lesions after 14 days of elacestrant treatment compared to baseline.

Overall, all three studies focused on elacestrant as a potential treatment for hormone receptor-positive breast cancer. Despite their differing designs and objectives, they collectively aimed to assess the efficacy and safety of elacestrant across various patient populations and treatment settings. These studies provided crucial insights into elacestrant as a novel SERD and its possible role in treating hormone receptor-positive advanced breast cancer.

### Post-hoc findings

**Figure 2:**
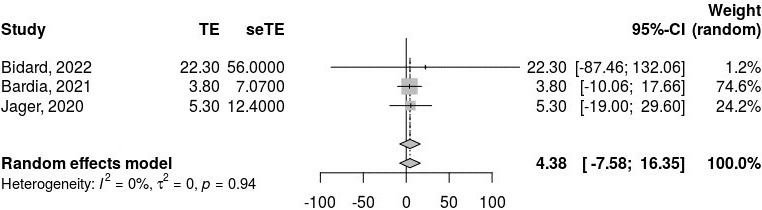
**Pooled progression-free survival (PFS) outcomes from the three combined studies using a random-effects model, revealing no significant difference in PFS outcomes and no heterogeneity among the included studies**. *I*^2^: inconsistency index; τ^2^: between-study variance; CI: confidence interval; TE: treatment effect/effect estimate; seTE: standard error of the treatment effect

We analyzed PFS outcomes from three combined trials. The pooled PFS based on the random-effects model was estimated at 4.38 (95% CI: –7.58 to 16.35; Figure 2). However, the results were not statistically significant (*z* = 0.72, *P* = 0.47), indicating that the intervention did not show a significant effect on PFS. The heterogeneity analysis demonstrated no τ^2^, with a τ^2^ value of 0, and the test for heterogeneity showed a Q-value of 0.11 with 2 degrees of freedom (*P* = 0.94), suggesting no heterogeneity among the studies.

**Figure 3:**
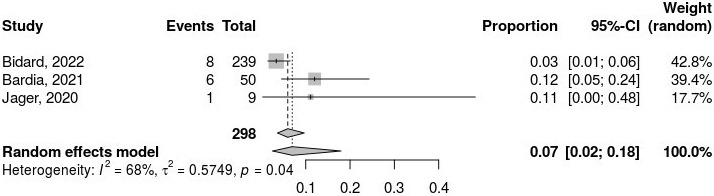
**Pooled proportion analysis of objective response rate (ORR) outcomes**. The meta-analytical method applied consists of the inverse variance method, DerSimonian-Laird estimator for τ^2^, logit transformation, and Clopper-Pearson confidence intervals for individual studies. *I*^2^: inconsistency index; τ^2^: between-study variance; CI: confidence interval

The pooled ORR was 7% (95% CI: 2% to 18%; Figure 3), based on the random-effects model. Heterogeneity analysis revealed considerable variability among the studies, with a τ^2^ value of 0.5749, an H-value of 1.78 (95% CI: 1.00 to 3.30), and an *I*^2^ statistic of 68% (95% CI: 0.0% to 90.8%). The Rb value was 63.6% (95% CI: 4.7% to 100.0%). The test for heterogeneity yielded a Q-value of 6.33 with 2 degrees of freedom (*P* = 0.04), confirming significant heterogeneity among the included studies.

### Ongoing clinical trials

Table 2 summarizes ongoing/unreported trials related to elacestrant, including their identifiers, interventions, outcome measures, participant counts, completion dates, and locations. Seven trials with various aims are outlined, including studies focused on CDK4/6 inhibitor-naive ER+, HER2– metastatic breast cancer, safety, and efficacy of elacestrant in combination with other drugs, and preoperative settings. These trials are in different stages, such as not yet recruiting, recruiting, active but not recruiting, and completed. They involve diverse interventions and outcome measures, such as PFS, ORR, duration of response, clinical benefit rate, and AE. The number of participants varies across the trials, ranging from 23 to 322, and the completion dates span from February 2022 to May 2030. The trials are being conducted in the United States, Belgium, Greece, and Spain.

**Table 2:** Identifier, interventions, outcome measures, participants, completion date and locations of ongoing/unreported trials

**No.**	**NCT**	**Title**	**Status**	**Conditions**	**Interventions**	**Outcome measures**	**Phase**	***N***	**Study Design**	**Completion Date**	**Collaborators**	**Locations**
1	NCT05596409	ELACESTRANT in Women and Men With CDK4/6 Inhibitor-Naive Estrogen Receptor Positive, HER-2 Negative Metastatic Breast Cancer Study (ELCIN)	Not yet recruiting	Metastatic breast cancer	Elacestrant	PFS; ORR; DOR; clinical benefit rate; PFS; OS	2	80	Single group, open label, interventional	August, 2025	Stemline Therapeutics, Inc.	United States
2	NCT05563220	Open-Label Umbrella Study To Evaluate Safety And Efficacy Of Elacestrant In Various Combination In Patients With Metastatic Breast Cancer (ELEVATE)	Recruiting	Breast cancer; metastatic breast cancer	Elacestrant; alpelisib; everolimus; ribociclib; palbociclib	RP2D; safety; pharmacokinetic assessment profile; ORR; DOR; clinical benefit rate; PFS; OS	1 & 2	322	Non-randomized, parallel assignment, open label, interventional	August, 2026	Stemline Therapeutics, Inc.	United States
3	NCT05618613	Study of Elacestrant in Combination With Onapristone in Patients With Advanced or Metastatic Breast Cancer (ELONA)	Active, not recruiting	Breast cancer	Elacestrant; onapristone	RP2D; ORR per RECIST version 1.1.; AE, SAE, changes in clinical laboratory values; vital sign measurements; changes in ECG parameters; area under the plasma concentration-time curve over the dosing interval; Cmax, Tmax; trough concentration; clinical benefit rate; PFS; OS	1 & 2	67	Single group, open label, interventional	April, 2026	Context Therapeutics Inc.	United States
4	NCT04791384	Phase Ib/II Trial of Abemaciclib and Elacestrant in Patients With Brain Metastasis Due to HR+/Her2- Breast Cancer	Recruiting	Breast cancer	Abemaciclib; elacestrant	AE; efficacy of the combination abemaciclib and elacestrant; tumor response rates; duration of tumor response rates; completion rate	1 & 2	44	Sequential assignment, open label, interventional	June, 2023	Criterium, Inc.	United States
5	NCT05512364	TREAT ctDNA Elacestrant	Not yet recruiting	ER+ breast cancer; HER2– breast cancer; stage IIB breast cancer; stage III breast cancer	Elacestrant; tamoxifen; letrozole 2.5 mg; anastrozole 1 mg; exemestane 25 mg	DMFS; ctDNA elimination rate at month 1	3	220	Randomized, parallel assignment, open label, interventional	May, 2030	European Organisation for Research and Treatment of Cancer - EORTC; Breast International Group; Menarini Group	Belgium, Greece
6	NCT05386108	Study of Abemaciclib and Elacestrant in Patients With Brain Metastasis Due to HR+/HER2- Breast Cancer (ELECTRA)	Recruiting	Breast neoplasms; breast diseases	Elacestrant; abemaciclib	RP2D; ORR; intracranial response rate per RECIST; intracranial response rate per RANO; duration of tumor response; clinical benefit rate; duration of PFS	1 & 2	106	Non-randomized, sequential assignment, open label, interventional	December, 2025	Stemline Therapeutics, Inc.	United States, Korea, Spain
7	NCT04797728	Elacestrant in Preoperative Setting, a Window of Opportunity Study (ELIPSE)	Completed	Breast cancer; hormone receptor positive breast carcinoma	Elacestrant	Complete cell cycle arrest; PAM50 (prediction analysis of microarray 50) subtype change; gene expression change; AE; global gene expression changes; gene expression-based signature of response; CelTIL score; mean change in Ki67; changes in the distribution of somatic mutations; ctDNA	Early, 1	23	Single group, open label, interventional	February, 2022	SOLTI Breast Cancer Research Group; Radius Health, Inc.	Spain

AE: adverse events; OS: overall survival; PFS: progression-free survival; RP2D: recommended phase 2 dose; ORR: objective response rate; HER2–: HER2-negative; ER+: estrogen receptor-positive

## Discussion

The current evidence supporting the use of elacestrant in patients with advanced breast cancer has led to the initiation of several other clinical trials. These trials are exploring the use of elacestrant in early-stage breast cancer and its combination with other therapies for metastatic cases, including drugs like alpelisib, everolimus, and CDK4/6 inhibitors [19]. The research includes two phase I trials and one phase III trial (EMERALD), which revealed significant insights into disease progression and patient survival, though no conclusive results were achieved. The phase I trials specifically targeted ER+, HER2– breast cancer, with the objective of evaluating elacestrant’s safety, tolerability, and preliminary efficacy. However, the small sample sizes may limit the broader applicability of the findings. A post-hoc analysis of PFS outcomes across these trials showed no statistically significant benefit for the intervention group. While the uniformity across the studies suggests methodological consistency and similar patient populations, this does not confirm the efficacy of elacestrant. The ORR was estimated at 0.07, with a 95% confidence interval ranging from 0.02 to 0.18, pointing to a relatively low response rate. Nevertheless, due to the variability and limited scope of the available data, it remains difficult to draw a definitive conclusion regarding the effectiveness of elacestrant in treating breast cancer.

The phase III EMERALD trial highlighted encouraging outcomes for elacestrant in the management of ER+/HER2– metastatic breast cancer [20]. The study showed a significantly extended PFS for patients receiving oral elacestrant compared to those on standard care, particularly in individuals whose disease had progressed after prior endocrine therapy and CDK4/6 inhibitor treatment [21]. However, its efficacy in patients who have not received fulvestrant remains unclear, emphasizing the need for further research. Elacestrant’s pharmacological profile is complex, exhibiting dose-dependent ER antagonist and agonist effects, along with the ability to penetrate the blood-brain barrier (BBB) [20]. This feature could be significant in treating breast cancer patients with CNS involvement.

In the phase III EMERALD trial, patients with *mESR1*-mutant tumors experienced a 45% reduction in the risk of disease progression or death when treated with elacestrant compared to standard endocrine therapy. In this group, the median PFS was 3.79 months with elacestrant, compared to 1.87 months for those receiving standard treatment (HR, 0.54; *P* < 0.001) [22]. Elacestrant also showed higher PFS rates at both 6 months (34.3% vs. 20.4%) and 12 months (22.3% vs. 9.4%), indicating sustained benefits from this oral SERD therapy. The improved PFS was notably consistent among patients with *mESR1* mutations. Specifically, in this subgroup, the PFS rates at 6 months were 40.8% for elacestrant compared to 19.1% for standard therapy, and at 12 months, they were 26.8% vs. 8.2% [22]. Additionally, subgroup analysis from the EMERALD trial revealed that elacestrant provided clinical benefits for patients who had previously received fulvestrant, regardless of their *ESR1* mutational status, highlighting its potential as a treatment option for refractory HR+ breast cancer [23].

Elacestrant, a nonsteroidal SERD, exhibits dose-dependent ER antagonist and agonist activities, positioning it as a leading candidate for treating HR-positive breast cancer [24]. Studies have shown that elacestrant can act as an ER agonist within the CNS and readily crosses the BBB [25]. Laboratory research with doses ranging from 0.3 mg/kg to 100 mg/kg has indicated no significant effects on uterine wet weight or epithelial thickness [26]. However, at a low dose of 0.3 mg/kg, a notable increase in uterine weight was observed, whereas no such effect occurred at doses of 1 mg/kg or higher [25]. This suggests that elacestrant’s activity may shift towards antagonism at higher doses [25]. In both single-ascending dose and multiple-ascending dose trials, doses up to 1,000 mg daily were found to be safe and well tolerated, with no maximum tolerated dose identified. Elacestrant’s oral bioavailability was approximately 10%, with a half-life ranging from 27 h to 47 h, reaching steady-state levels after 5–6 days of administration. After seven days of treatment, the mean ER occupancy in the uterus was 83% at 200 mg and 92% at 500 mg daily. The median ratio of elacestrant in cerebrospinal fluid compared to plasma was 0.126% for the 500 mg dose and 0.205% for the 200 mg dose [27]. These complex pharmacological properties may influence how elacestrant is used in the treatment of breast cancer in the future.

Reported side effects of elacestrant include hypercholesterolemia and hypertriglyceridemia, occurring in 30% and 27% of patients, respectively. The rates of severe (Grade 3 and 4) hypercholesterolemia and hypertriglyceridemia were relatively low, at 0.9% and 2.2%, respectively [28]. Other more frequently observed side effects include musculoskeletal pain, nausea, and various gastrointestinal issues [29]. Patients taking elacestrant are also advised against breastfeeding due to potential risks [29]. However, due to the limited ability of elacestrant and other SERDs to effectively cross the BBB, breast cancer patients with brain metastases are still unable to fully benefit from SERD treatments [30].

The findings from this post-hoc analysis suggest a relationship between the effectiveness of ER-targeting therapies and the levels of ER and PR expression in tumor cells. High ER expression enhances the interaction with SERDs, while PR expression indicates a reliance on ER signaling for tumor growth and survival [11]. This is significant because current oncology guidelines recommend endocrine therapy for ER+ tumors, even when ER is expressed in as few as 1% of tumor cells [31]. Upcoming clinical trials that will compare elacestrant with fulvestrant as the foundational endocrine therapy, in combination with treatments like CDK4/6 inhibitors, alpelisib, and everolimus, are expected to provide valuable insights into the best SERD for second-line treatment [32]. Based on this information, the effectiveness of endocrine therapies in breast cancer treatment appears to be closely tied to the expression of ER and PR within tumors [33]. However, to determine the most suitable endocrine therapy, whether SERD or another type, further research and clinical trials are required to assess their efficacy and safety fully [34]. Ultimately, the selection of treatment should be individualized, taking into consideration the tumor’s characteristics and the patient’s overall health, with input from a multidisciplinary team of healthcare professionals [35].

### Conclusion

Although elacestrant has been approved for use in patients who have undergone at least one round of endocrine therapy, our study highlights the need for additional clinical research to explore its potential in various contexts, such as treating endocrine-naive patients or those who have received extensive treatment for ER+ breast cancer. While phase I trials and post-hoc analyses show promise for elacestrant as a treatment for ER+, HER2– breast cancer, the current evidence is not yet strong enough to confirm its effectiveness. Studies with larger sample sizes and more comprehensive phase II and III trials are needed to establish elacestrant's definitive role in treatment of patients with breast cancer.

## Abbreviations

AE: adverse events
BBB: blood-brain barrier
BICR: blinded independent central review
ER+: estrogen receptor-positive
HER2–: HER2-negative
ORR: objective response rate
OS: overall survival
PFS: progression-free survival
RP2D: recommended phase 2 dose
SERD: selective estrogen receptor degrader
SOC: standard of care
τ^2^: between-study variance

## Declarations

### Availability of data and materials

All data utilized for the purpose of this study are available publicly and online.

### Copyright

© The Author(s) 2025.

## References

[1] Shah N, Mohammad AS, Saralkar P, Sprowls SA, Vickers SD, John D (2018). Investigational chemotherapy and novel pharmacokinetic mechanisms for the treatment of breast cancer brain metastases. Pharmacol Res.

[2] Lim E, Metzger-Filho O, Winer EP (2012). The natural history of hormone receptor-positive breast cancer. Oncology (Williston Park).

[3] Giordano SH, Buzdar AU, Hortobagyi GN (2002). Breast cancer in men. Ann Intern Med.

[4] Elayoubi J, Chi J, Mahmoud AA, Alloghbi A, Assad H, Shekhar M (2023). A Review of Endocrine Therapy in Early-stage Breast Cancer: The Journey From Crudeness to Precision. Am J Clin Oncol.

[5] Al-Qasem AJ, Alves CL, Ditzel HJ (2021). Resistance Mechanisms to Combined CDK4/6 Inhibitors and Endocrine Therapy in ER+/HER2- Advanced Breast Cancer: Biomarkers and Potential Novel Treatment Strategies. Cancers (Basel).

[6] Lainé M, Fanning SW, Chang YF, Green B, Greene ME, Komm B (2021). Lasofoxifene as a potential treatment for therapy-resistant ER-positive metastatic breast cancer. Breast Cancer Res.

[7] Sanchez KG, Nangia JR, Schiff R, Rimawi MF (2022). Elacestrant and the Promise of Oral SERDs. J Clin Oncol.

[8] Li Y, Orahoske CM, Urmetz SM, Zhang W, Huang Y, Gan C (2022). Identification of estrogen receptor down-regulators for endocrine resistant breast cancer. J Steroid Biochem Mol Biol.

[9] Pancholi S, Simigdala N, Ribas R, Schuster E, Leal MF, Nikitorowicz-Buniak J (2022). Elacestrant demonstrates strong anti-estrogenic activity in PDX models of estrogen-receptor positive endocrine-resistant and fulvestrant-resistant breast cancer. NPJ Breast Cancer.

[10] Patel HK, Bihani T (2018). Selective estrogen receptor modulators (SERMs) and selective estrogen receptor degraders (SERDs) in cancer treatment. Pharmacol Ther.

[11] Bihani T, Patel HK, Arlt H, Tao N, Jiang H, Brown JL (2017). Elacestrant (RAD1901), a Selective Estrogen Receptor Degrader (SERD), Has Antitumor Activity in Multiple ER^+ ^Breast Cancer Patient-derived Xenograft Models. Clin Cancer Res.

[12] ORSERDU^TM^ (elacestrant) tablets, for oral use Initial U.S. Approval: 2023 [Internet]. https://www.accessdata.fda.gov/drugsatfda_docs/label/2023/217639s000lbl.pdf.

[13] Shah M, Lingam H, Gao X, Gittleman H, Fiero MH, Krol D (2024). US Food and Drug Administration Approval Summary: Elacestrant for Estrogen Receptor-Positive, Human Epidermal Growth Factor Receptor 2-Negative, *ESR1*-Mutated Advanced or Metastatic Breast Cancer. J Clin Oncol.

[14] Foong SYF, Simpson PT, Cummings MC, Lakhani SR, Shin SJ, Chen YY, Ginter PS Future Role of Molecular Profiling in Small Breast Samples and Personalised Medicine. A Comprehensive Guide to Core Needle Biopsies of the Breast.

[15] Page MJ, McKenzie JE, Bossuyt PM, Boutron I, Hoffmann TC, Mulrow CD (2021). The PRISMA 2020 statement: An updated guideline for reporting systematic reviews. PLoS Med.

[16] Bidard FC, Kaklamani VG, Neven P, Streich G, Montero AJ, Forget F (2022). Elacestrant (oral selective estrogen receptor degrader) Versus Standard Endocrine Therapy for Estrogen Receptor-Positive, Human Epidermal Growth Factor Receptor 2-Negative Advanced Breast Cancer: Results From the Randomized Phase III EMERALD Trial. J Clin Oncol.

[17] Bardia A, Kaklamani V, Wilks S, Weise A, Richards D, Harb W (2021). Phase I Study of Elacestrant (RAD1901), a Novel Selective Estrogen Receptor Degrader, in ER-Positive, HER2-Negative Advanced Breast Cancer. J Clin Oncol.

[18] Jager A, de Vries EGE, der Houven van Oordt CWM, Neven P, Venema CM, Glaudemans AWJM (2020). A phase 1b study evaluating the effect of elacestrant treatment on estrogen receptor availability and estradiol binding to the estrogen receptor in metastatic breast cancer lesions using ^18^F-FES PET/CT imaging. Breast Cancer Res.

[19] Varella L, Cristofanilli M (2023). Evaluating Elacestrant in the Management of ER-Positive, HER2-Negative Advanced Breast Cancer: Evidence to Date. Onco Targets Ther.

[20] Olivier T, Prasad V (2022). Elacestrant in metastatic breast cancer: Is the “standard of care” meeting standard requirements?. Transl Oncol.

[21] Kaklamani VG, Bidard FC, Neven P, Montero AJ, Mouret-Reynier MA, Sohn J (2023). Oral elacestrant vs standard-of-care in estrogen receptor-positive, HER2-negative (ER+/HER2-) advanced or metastatic breast cancer (mBC) without detectable ESR1 mutation (EMERALD): Subgroup analysis by prior duration of CDK4/6i plus endocrine therapy (ET). J Clin Oncol.

[22] Jacobson A (2022). Elacestrant Improves Progression-Free Survival After Endocrine Therapy for Estrogen Receptor-Positive Metastatic Breast Cancer. Oncologist.

[23] Dubash TD, Bardia A, Chirn B, Reeves BA, LiCausi JA, Burr R (2023). Modeling the novel SERD elacestrant in cultured fulvestrant-refractory HR-positive breast circulating tumor cells. Breast Cancer Res Treat.

[24] Chen YC, Yu J, Metcalfe C, De Bruyn T, Gelzleichter T, Malhi V (2022). Latest generation estrogen receptor degraders for the treatment of hormone receptor-positive breast cancer. Expert Opin Investig Drugs.

[25] Wardell SE, Nelson ER, Chao CA, Alley HM, McDonnell DP (2015). Evaluation of the pharmacological activities of RAD1901, a selective estrogen receptor degrader. Endocr Relat Cancer.

[26] Garner F, Shomali M, Paquin D, Lyttle CR, Hattersley G (2015). RAD1901: a novel, orally bioavailable selective estrogen receptor degrader that demonstrates antitumor activity in breast cancer xenograft models. Anticancer Drugs.

[27] Conlan MG, de Vries EFJ, Glaudemans A, Wang Y, Troy S (2020). Pharmacokinetic and Pharmacodynamic Studies of Elacestrant, A Novel Oral Selective Estrogen Receptor Degrader, in Healthy Post-Menopausal Women. Eur J Drug Metab Pharmacokinet.

[28] (2023). Elacestrant Dihydrochloride. Am J Health Syst Pharm.

[29] Beumer JH, Foldi J (2023). Pharmacology and pharmacokinetics of elacestrant. Cancer Chemother Pharmacol.

[30] Zhou F, Yang G, Xue L, Liu Y, Guo Y, Zhu J (2023). SCR-6852, an oral and highly brain-penetrating estrogen receptor degrader (SERD), effectively shrinks tumors both in intracranial and subcutaneous ER + breast cancer models. Breast Cancer Res.

[31] Hammond ME, Hayes DF, Dowsett M, Allred DC, Hagerty KL, Badve S, American Society of Clinical Oncology, College of American Pathologists (2010). American Society of Clinical Oncology/College of American Pathologists guideline recommendations for immunohistochemical testing of estrogen and progesterone receptors in breast cancer (unabridged version). Arch Pathol Lab Med.

[32] Hoy SM (2023). Elacestrant: First Approval. Drugs.

[33] Zhong Y, Ding B, Qian L, Wu W, Wen Y (2022). Hormone Receptor Expression on Endocrine Therapy in Patients with Breast Cancer: A Meta-Analysis. Am Surg.

[34] Wang Y, Tang SC (2022). The race to develop oral SERDs and other novel estrogen receptor inhibitors: recent clinical trial results and impact on treatment options. Cancer Metastasis Rev.

[35] Rostoft S, van den Bos F, Pedersen R, Hamaker ME (2021). Shared decision-making in older patients with cancer - What does the patient want?. J Geriatr Oncol.

